# The nurse without a nurse: the antecedents of presenteeism in nursing

**DOI:** 10.1186/s12912-021-00669-1

**Published:** 2021-08-13

**Authors:** Mohammad Mehdi Mohammadi, Nahid Dehghan Nayeri, Shokoh Varaei, Arezoo Rasti

**Affiliations:** 1 Department of Medical Surgical Nursing, School of Nursing & Midwifery, Tehran University of Medical Sciences, Tehran, Iran; 2 Nursing and Midwifery Care Research Center, School of Nursing and Midwifery, Tehran University of Medical Sciences, Tehran, Iran

**Keywords:** Presenteeism, Nursing, Concept formation, Qualitative study

## Abstract

**Background:**

Presenteeism describes the state in which staff who lack the conditions for being present at work and need rest and leave for various reasons (such as illness, low spirits, fatigue, etc.) are present at the workplace. Due to the lack of knowledge about the antecedents of presenteeism in nurses and the context-based nature of this concept, the present study was conducted to explain the reasons for presenteeism in nurses.

**Methods:**

This qualitative study was performed using the qualitative content analysis method. The study population consisted of 17 nurses working in different wards of hospitals. In this regard, data were collected from February to June 2020 using individual, face-to-face, in-depth, semi-structured interviews and were analyzed using qualitative content analysis.

**Results:**

The nurse without a nurse was a category introduced as an antecedent of presenteeism. In this respect, nurses experienced limited power, injustice, compulsory presence, inadequate structural facilities, damaged professional identity, manager-nurse disconnect, insufficient knowledge, physical and mental health complications, job stress, job burnout, multitasking, and impaired communication.

**Conclusion:**

The nurse, who has been responsible for caring, supporting, advising, advocating, and educating the patient, has now been left without a nurse. In other words, not nursing the nurse has given rise to the emergence of presenteeism. It is recommended that the results of this study be used in making health policies. The results of this study can make nurses’ voices heard by health leaders and managers. A voice that has never been heard as it deserves.

**Supplementary Information:**

The online version contains supplementary material available at 10.1186/s12912-021-00669-1.

## Background

Presenteeism is defined as the physical presence of employees in the workplace when they are unable to be fully engaged and productive in the work environment [1]. This means that some employees should not be present at work due to some reasons, but they are overly present at work [2].

Organizations that are affected by presenteeism will face reduced productivity and lack of innovation [3, 4]. The physical presence of a person along with his/her problems in the workplace reduces his/her productivity at work, as evidence suggests that presenteeism almost reduces productivity four times more than absenteeism [5, 6]. On the other hand, the economic costs imposed by presenteeism are also of paramount importance. For instance, $ 25 billion to $ 34 billion a year in Australia and $ 150 billion to $ 250 billion a year in the United States are spent on presenteeism [5, 7].

Presenteeism is more common in occupations that require high social and interpersonal communication skills, such as nursing. Nurses are among the health care groups that spend more time and have the most contact and interaction with clients [8, 9]. Presenteeism among nurses gradually leads to the collapse of the health organization. Consequently, problems such as reduction of productivity and job satisfaction, increasing of human errors and job burnout, and many other unknown consequences can occur [10–12].

The complexity, multidimensionality, and breadth of the concept of presenteeism have led to the failure to present the antecedents of this concept in a complete and comprehensive way. However, it is not possible to deal with a destructive phenomenon without explaining and identifying its antecedents accurately. On the other hand, a comprehensive explanation of the antecedents of presenteeism in nursing paves the way for the identification of this concept in a profession that deals with health as the most valuable human capital [13]. It is obvious that concepts are defined according to social contexts and a concept can have various meanings in different contexts. Therefore, the context in which a concept occurred should be examined in order to achieve the antecedents of that concept [14]. Since presenteeism is influenced by the managerial, structural, social, and organizational culture contexts, the context in question must be considered in order to explain presenteeism antecedents [13]. With this in mind, there is a need to explain the antecedents of presenteeism based on the live experiences of the participants.

In our previous study [13], we identified attributes of presenteeism. According to the results of our previous study, there are three cognitive, emotional, and physical movement capacities in each nurse, and the impairment of each in the workplace can lead to an experience of the presenteeism by the nurse. In cognitive impairment, the nurse with presenteeism has difficulty processing information and concentrating at work, leading to disruption of tasks that were previously performed well [15]. Moreover, the nurse’s mental processing slows down and his/her power of predicting future situations in caring decreases [16]. Important principles that must be observed in caring for a client are forgotten and he or she has difficulty remembering the care provided [16–18]. In emotional impairment, what emanates from emotion, art, tenderness, and the spirit of caring does not manifest itself in the workplace. The nurse with presenteeism ignores the client’s spirit while caring for him/her as if he/she is in contact with and responsible for caring for an object [18–21]. This nurse does not have an understanding of the client’s vulnerability and does not see what is important to the client [21–23]. Kim et al., equated presenteeism with the loss of nursing spirit in which the nurse’s compassion is not given a chance to be manifested, the nurse feels that her mental energy is drained, and she feels a kind of extreme emotional exhaustion that gradually leads to the insensitivity of the care provided by him/her [20]. Impairment of the nurse’s emotional capacity disrupts the nurse’s sense of love for the client, and the nurse’s self-sacrificing force, which results from love for the client, is faced with extreme fatigue [19]. The extraversion of emotions is suppressed in the nurse with presenteeism and her/his sense of pleasure, happiness, and humor is less felt [13]. Contrary to the cognitive and emotional characteristics of presenteeism, which are in relation with the mind and soul, the nurse’s physical capacity and function are affected in physical movement impairment. The caring speed and accuracy of the nurse will be reduced and his/her previous stability and balance will be disappeared [5, 24]. In the present study, we sought to find out what antecedents can lead to the impairment of any of these capacities and thus ultimately bring about presenteeism in the nurses.

Given that so far no study in Iran has examined the antecedents of presenteeism in nursing, it seems necessary to explore the antecedents of presenteeism in this profession taking into account the context of Iran. Accordingly, the present study aimed to identify the antecedents of presenteeism among nurses in Iran.

## Methods

### Design

This study was a part of the dissertation performed in partial fulfillment of the requirements for the degree of Ph.D. in Nursing. In this study, the qualitative method and in-depth interviews were used. Qualitative methodology in the present study helped gain insights into the antecedents of presenteeism in nurses through real-life descriptions and narrations. Accordingly, the experiences embedded and formed in the social context were extracted. According to Rogers, in the present study, antecedents are phenomenal found to precede an instance of the concept [14]. The study protocol was approved by Tehran University of Medical Sciences (TUMS) before data collection began (# 9711199002).

### Participants

The main study population consisted of 17 nurses working in different wards of the hospital, including the emergency department, intensive care unit, oncology ward, medical-surgical unit, and the psychiatric ward. A purposeful sampling method was used to recruit participants from teaching hospitals in Iran. Data saturation determined the sample size. This implies that the interviews were stopped merely when, after analysis of at least ten interviews, new data were not observed in three consecutive interviews [25]. Inclusion criteria included the nurse’s employment in the hospital, the nurse’s direct involvement in the patient care, and having at least 1 year of work experience.

As the study progressed, participants were selected based on increasing maximum diversity (in terms of sex, level of education, age, type of unit, work experience).

### Data collection

Data of the present study were collected from February to June 2020. The main data collection method was individual, face-to-face, in-depth, semi-structured interviews. All interviews were conducted by the first author (MMM), a male Ph.D. student in nursing. MMM was already trained in ethical communication practices and interviewing techniques. The interviews were conducted in Persian, and the average duration of each interview was 70 min (ranging from 45 to 90 min). Totally, 23 interviews were conducted, and in this regard, 6 participants were interviewed twice. Interviewing 6 participants twice was due to the fact that after the initial analysis of the interviews, further questions arose and it was necessary to ask them during the second interview.

Interviews were audio-recorded and transcribed verbatim. Participants had no prior knowledge of the study, the interviewer, and the study objectives. Demographic information (age, sex, marital status, education level, and work experience) and contact information (mobile number and e-mail) of the participants were collected through a short questionnaire. Interviews began with guiding questions (“Have you ever been present at work when you were not ready?”, “Please describe your experience of being at work despite your illness”, “Please describe reasons why you are still at work despite the problems you have.”) and were directed with probing questions (“Please explain more about the subject, and if possible, give an example.”, “What happened next?”, “What do you mean by that?”, and “Is there another aspect that we did not discuss during the interview, or what would you like to explain more about?” Although the guiding questions were used in the present study, there was no fixed order of questions to ask. All participants chose the hospital they worked in as their interview setting, and the interviews were conducted in a quiet, well-lit room. The time for conducting the interviews, based on the participants’ choice, was after the end of their work shift.

### Ethical considerations

This study was ethically approved by the ethics committee of School of Nursing and Midwifery and Rehabilitation, Tehran University of Medical Sciences (IR.TUMS.FNM.REC.1398.189). The study was found to be in accordance to the ethical principles of the Declaration of Helsinki [26]. All participants were informed about the study process and its objectives, and the written informed consent was obtained from them. The permission to audio-record the interviews was also obtained, and participants were assured about confidentiality and anonymity of the collected data. Participants had the right to withdraw from the study at any stage. However, no participants refused to participate in the study or dropped out of the study after enrollment.

### Data analysis

In this study, the conventional content analysis proposed by Graneheim and Lundman was used for data analysis [27]. The reason for using this type of analysis in the present study was the lack of sufficient knowledge and theory about the concept of presenteeism in Iran. Therefore, it was possible to extract categories directly from data obtained through interviews without considering pre-defined categories. Each step of data analysis process in the present study is shown in Table 1. Microsoft Word 2007 (Microsoft Corp., Redmond WA) software was used to transcribe the interview texts, and MAXQDA 2010 (VERBI Software GmbH, Berlin, Germany) software was used to manage and analyze the data.

**Table 1:** Data analysis steps and actions taken

levels		Actions taken
Step 1	Transcribing interviews	After each interview, the first author (MMM) listened to the recorded audio file within 24 h and transcribed it verbatim into Persian.
Step 2	Gaining general sense	Each interview text was read and re-read to make sense of the data in general to make sense of the data.
Step 3	Recording and reviewing interpretive notes	To understand the meaning of the data, the initial ideas were recorded and reviewed in interpretive notes.
Step 4	Identification of units of analysis and their coding	Sentences and paragraphs that were meaningful in relation to the research questions were identified and inductively coded.
Step 5	Formation of categories and subcategories	The codes were compared based on differences and similarities and sorted into emerging categories and subcategories. The formed codes and classes were reviewed by the second and third authors (ND and SV) until a consensus was reached.
Step 6	Translation	After the final results emerged, the text was translated from Persian into English by an external translator.
Step 7	Reviewing and modifying translated text	The translated text was reviewed by MMM, and final modifications and consensus were reached.

### Data trustworthiness

Lincoln and Guba (1985) introduced four criteria of credibility, dependability, confirmability, and transferability to assure the trustworthiness of qualitative studies [28]. We took some measures to enhance the trustworthiness of our data. The details are shown in Table 2.

**Table 2:** Measures taken to increase data trustworthiness

Criterion	Measures
Credibility	Member checking, prolonged engagement with the research setting, and maximum variation sampling
Dependability	Recording and transcribing all the interviews word by word, analyzing the data immediately after collecting them, and using participants’ quotes
Confirmability	All stages of the research were recorded, enabling others to review and retrieve them.
Transferability	A rich description of participants’ characteristics and their experiences

## Results

Participants of the present study included 17 nurses (10 females (58.83%) and 7 males (41.17%)). The mean (standard deviation) age of the participants was 37.29 (6.85) years old, ranging from 30 to 52. In terms of marital status, 5 (29.42%) participants were single and 12 (70.58%) were married. Regarding education level, 3 (17.65%) had a master’s degree and 14 (82.35%) had a bachelor’s degree. The mean (standard deviation) of nurses’ work experience was 13 years (6.81), ranging from 6 to 28 years. Other characteristics of the participants are shown in Additional file 1.

### The nurse without a nurse

The nurse without a nurse is a category that has been introduced as the main antecedent of presenteeism. The nurse, who has been a supporter, advocate, educator, coordinator, counselor, and caregiver and the one who has spread all the goodness to the patient, has now been left without anyone performing these roles for her/him. As depicted from Table 3, the category of the nurse without a nurse was extracted. According to this table, the nurse who was the supporter, patient advocator, and coordinator, at the present faced with issues such as limited power, perceived injustice, compulsory presence, structure failure, damaged professional identity, and multitasking due to the lack of a coordinator, lawyer, advocator, and supporter. Even though the nurse had the role of a counselor in her/his career, she/he currently experienced mental health complications, impaired communication, job stress, and preoccupation with life due to the lack of having a compassionate counselor. The nurse, who was an educator for the patient and enhanced the patient’s knowledge, sometimes found himself or herself in a situation where he or she might be drawn to presenteeism due to insufficient knowledge. The nurse who cared for the patient’s physical illness, currently she/he had no one to alleviate his or her physical health complications. In other words, by “the nurse without a nurse”, we meant that the nurse, who had many roles in caring for the patient and supported the patient in a comprehensive way, had been abandoned by managers and others involved in incidence of presenteeism in nurses. It seems that the nurse needs others to take on the role of nurse for her/him.

**Table 3:** The antecedents of presenteeism in nursing

Category	Subcategories
***The nurse without a nurse***	*Limited power*
	*Perceived injustice*
	*Compulsory presence*
	*Inadequate structural facilities*
	*Damaged professional identity*
	*Manager-nurse disconnect*
	*Insufficient knowledge*
	*Physical health complications*
	*Mental health complications*
	*Preoccupation with life*
	*Job stress*
	*Occupational burnout*
	*Multitasking*
	*Impaired communication*

### Limited power

One of the issues that the participants mentioned as an example of powerlessness and the antecedent of presenteeism was lacking authority to use theoretical science. In other words, in this case, the nurse, despite having theoretical knowledge, will not be allowed to use their knowledge in the clinical field, and this will lead to their annoyance at work. One of the participants stated in this regard:

*“We, as nurses, aren’t allowed to use many of the courses we’ve taken at university. Every time I come across a subject that I know, but don’t have the power to use, I get nervous. When I see that I’m so powerless that I can’t even use my own knowledge, I lose my motivation” (P # 10).*

Sometimes the nurse feels that no strategic thinking is assumed for them and that they are merely service providers; A person who provides services, does not require thinking, has no power to change, and is obliged to be the sole executor of the order. One of the participants stated in this regard:

*“As a nurse, I feel that the treatment decision made for the patient will ultimately lead to the patient’s death! But we have no place to change the treatment process! It’s as if we just have to follow orders, like a robot” (P # 12).*

Nurses have limited power so that they will be convicted in the event of an error. As a result, they imagine themselves in an unstable situation in which a problem will arise, and they will be convicted. The experiences of the participants in this regard are as follows:

*“It’s as if we’re walking in a minefield! A powerless creature that’s thrown into a minefield! If you make only one mistake, you’re totally condemned and forced to pay a ransom” (P # 4).*

### Perceived injustice

Based on the experiences of nurses, injustice is one of the issues that lead to the emergence of presenteeism in them since nurses suffer emotionally in an environment full of injustice, and the imbalance of presence appears in them. The nurse, who is the patient’s advocate and supporter, now needs an advocate to nurse him/her.

Various experiences have shown that there is no justice in giving a chance to leave and rest, which will be described below:

*“Sometimes, you see a series of injustice in the nursing system that makes you mad at work. Someone is the head nurse’s pet and can take time off from work, but we can’t have it” (P # 7).*

Injustice in promotion is another antecedent mentioned by the participants. They discussed the closure of the promotion path, the lack of a clear mechanism and fair process for promotion, and disregard for the efforts and education in promotion:

*“A nurse is like a soldier whose commander is the doctor! A subordinate soldier who has no way to be promoted!” (P # 9).*

The issue of injustice in the payment of salaries and perks was raised by nurses. In this regard, nurses, on the one hand, face a disproportionate amount of wages to the tasks performed, and, on the other hand, there is no real justice between the salary of a doctor and a nurse in comparison. Participants stated in this regard:

*“As if there’s no justice. Compare a doctor’s income with a nurse’s! I have a lot of installments, and I need money. I really need to get a lot of shifts, so I can repay my expenses” (P#2).*

### Compulsory presence

Participants noted that sometimes their presence was due to the compulsion imposed on them in various ways. Organizational policies are forced to impose a kind of obligatory presence on the nurse due to problems such as lack of nursing staff:

*“If we have a problem, we can’t take leave immediately because a reserve staff that can replace us quickly hasn’t already been determined, so the head nurse generally doesn’t agree with the nurse to take a leave” (P # 7).*

Managers of health organizations, including head nurses, play a significant role in imposing this obligation. They are directly involved in the presence, absence, and scheduling of nurses’ work shifts. The experiences of nurses in this regard are as follows:

*“Some medical staff and nursing managers think that compassionate care should only be given to hospitalized patients. And they treat completely emotionless and compulsorily about our problems, and when I ask the reason, they say: “hey lady, I can’t sacrifice all these sick people for your absence! You’re a nurse and have to come to your job!”” (P # 5).*

Financial needs impose more compulsion on the nurse. In other words, in case the nurse is not present at work, they will not be able to cover their living expenses:

*“Nursing can’t meet my financial needs. I’ve to take extra shifts, just to make ends meet. The salary I get doesn’t meet my financial needs” (P # 4).*

### Inadequate structural facilities

Structural facilities provide comfort and a sense of balance in nursing staff. Based on the participants’ experience, the inadequacy of these facilities in the health organization lead to the transmission of unpleasant feelings, fear, physical complications, and physical and mental fatigue.

The lack of emergency power systems and inappropriate nursing chairs in the hospital were among the inadequate structural facilities.

*“The hospital was old and didn’t have proper emergency power systems. During the night shifts, the power kept going out, and because I was a woman, I was afraid to check the rooms where the men were hospitalized” (P # 14).*

*“Chairs in the nursing station aren’t comfortable at all. The biggest part of the back pain I got at work was because of these chairs” (P # 9).*

Another participant pointed to the insufficient equipment to carry oxygen capsules:

*“There wasn’t essential equipment to move oxygen capsules in some of the wards I’ve worked in over the year. All of these factors have caused me to have back pain, and my back condition is getting worse every day” (P # 9).*

### Damaged professional identity

The damaged identity of nursing leads to frustration and discouragement of nurses in the workplace so that they become dispirited and disappointed by noticing public views on their field of study:

*“What always bothers me at work is that I feel we have no prestige in this profession; for example, doctors are much more prestigious and academic than their nursing colleagues” (P # 3).*

In addition to the damaged identity within the boundaries of the discipline, this identity is also recognized as impaired in the family and community based on the participants’ experience:

*“When I started studying nursing, my family used to say: ‘You’re a boy. You should’ve become a doctor, not a nurse. Nursing is for women’”.*

Nurses stated that in society, on the one hand, there is no distinction between academic and non-academic nursing, and, on the other hand, there is no independent identity for nursing:

*“I have a bachelor’s degree in nursing; it has occurred to me that when I say that I have a bachelor’s degree, people wonder if nursing also has a bachelor’s degree. Some people in the community think that nursing is a completely experimental job under the doctor’s supervision, and it makes me frustrated’” (P#9).*

### Manager-nurse disconnect

An antecedent of presenteeism, which was mainly emphasized by participants, was the disconnect between managers and nurses. It was as though an obstacle has been built between the nurse and the manager that prevents the manager from understanding, supporting, respecting, participating, and communicating properly with the nurse.

A participant mentioned the lack of trust and confidence in nurses:

*“Some time ago, I was in the middle of a shift when I felt sick; I was in a bad mood; I went to the locker room and lay down for a while. The supervisor came and reported on me because I was asleep! I just kept saying that I was sick and awful, she didn’t believe me. Then, I had to continue working with that bad situation” (P # 14).*

Another issue was incorrect evaluation. From the participants’ point of view, the method of evaluation by managers and purpose beyond it conveyed a sense of fear and insecurity to them:

*“Nursing managers secretly evaluate and reprimand us. You just have a stress all the time while working that someone is evaluating you now and wants to catch you red-handed!” (P # 6).*

Participants discussed the mistaken support of managers who follow organizational policies that encourage presence:

*“Our head nurses and supervisors not only don’t support a person who has a problem and comes to work despite having a problem but also encourage him/her to come to work more! Unfortunately, they wrongly believe that supporting these people is by appreciating them. While support here means allowing us to take time off” (P # 12).*

### Insufficient knowledge

Insufficient knowledge of the underlying conditions provides grounds for anxiety, concern, and confusion and leads to presenteeism. Thus, the person finds himself/herself under circumstances that they have not experienced before and is unknown. In addition, this limitation can stem from a lack of knowledge and skills and transmit a sense of inefficiency to the individual.

One issue that confronts an individual with new situations is the involuntary transfer to an unfamiliar ward:

*“When you’re transferred to a new ward, you’re afraid of making some mistakes, and if a critical situation arises in the ward because the department is unfamiliar, you’ll be more stressed” (P # 9).*

Moreover, the poor justification for employment laws and regulations, including leave laws, job descriptions, and other legal information, may lead to presenteeism:

*“When we entered the field of nursing, they didn’t explain to us how many times in a month we can take leave. If we get sick, can we take leave or not?” (P # 4).*

Lack of sufficient knowledge and skills in the face of new clinical conditions, particularly when it is urgently needed, provides grounds for anxiety and worry:

*“When I don’t have an experience of an emergency situation, and I don’t know what to do at that moment, I feel very bad, and I annoy myself for a long time why I didn’t do the right thing!” (P # 5).*

### Physical health complications

Physical health complications included illnesses, unhealthy lifestyles, unpleasant physical stimuli, and the like. According to the participants’ experiences, allergies and runny nose, asthma, migraine, food poisoning, diarrhea, vomiting, shoulder pain, knee pain, neck disc, headache, neck pain, toothache, and chronic diseases were among the diseases that could lead the nurse to presenteeism. The experience of one of the participants in this regard was as follows:

*“If the air is a little polluted, I breathe with difficulty. Going to work on that day is difficult for me because I have asthma” P (P # 9).*

Unpleasant physical stimuli are also associated with physical discomfort and cause confusion for the nurse. Accordingly, a physical stimulus can include an unpleasant sound, an annoying odor, an imbalanced temperature, and various other irritating factors received by sensory receptors:

*“The alarms of the dialysis devices get on my nerves; it may not be annoying for someone who comes to the ward for the first time, but if you work here for several years, you’ll understand what I’m saying” (P # 13).*

*“Nothing can sting me like the smell of a diabetic foot ulcer. From the beginning to the end of the dressing, despite wearing two masks, I’ve to constantly hold my breath to finish my work as quickly as possible with the least quality” (P # 11).*

Personal and professional lifestyles were also emphasized by the nurses:

*“We don’t sleep well; we don’t have eating habits or time to exercise. On the other hand, due to the night shifts, the body’s biological cycle is disrupted, and the sleep-wake cycle is unbalanced. When it’s night, you can’t fall asleep, but during the day, you’re drowsy” (P # 3).*

### Mental health complications

The complication is not exclusively physical but also mental. Anxiety and stress are among the factors leading to mental complications and consequently presenteeism. A participant’s experience in this regard was as follows:

*“A little of anxiety is good, but my problem is that sometimes I get overwhelmed, and I can’t do my nursing job properly. I’ve been taking 20 mg of propranolol before each shift for a long time” (P # 3).*

Grief and sorrow can be considered as other factors causing the nurse’s annoyance that lead to presenteeism:

*“Nothing in the ICU can upset me as much as the death of a young person! Seeing a young patient under ventilation, and when they’re end-stage, gets me down” (P # 11).*

In the meantime, complex emotional states such as fear were also discussed. The external source of this feeling, such as dark physical environment, patients with mental disorders, and fear of transmitting the disease was mentioned by the participants:

*“HIV-positive patients and infectious diseases like Corona gave me a sense of fear that I might get infected at work. This fear makes me unable to pass that sense of care to the patient as before” (P # 3).*

### Preoccupation with life

The preoccupation with nurses’ personal life and the issues occurring about their relatives continually have a significant impact on nurses’ presence. One of these issues that were repeatedly emphasized by the participants was concerns about their children:

*“I have a little girl that’s home alone and I worry a lot about her at work. I think by myself what if something happens to her on the way to school and a lot of other worries that give me the willies” (P # 8).*

Family disputes likewise lead to mental preoccupation and affect one’s performance in the workplace. As a result, there are constant conversations in the mind of the person who is reconstructing the situation of the dispute:

*“Once, I had an argument with my brother, and I went to work. If someone saw me at that moment, they’d actually think that I was at work and I was taking care of the patient, but they didn’t know what arguments and noises were in my mind.” (P # 15).*

Emotional involvement and being in love create an imbalance in one’s life. One of the participants stated:

*“I’d just fallen in love with my wife, who was in our ward. It had reached a point where my co-workers jokingly told me: What’s wrong with you? Are you in love? Why aren’t you on the ball?” (P # 6).*

### Job stress

This type of stress arises from the increase in energy demand in the profession. In this situation, the person faces a psychological burden in fulfilling his/her job responsibilities:

*“Our job is different from others. I’ve to respond to the patient’s needs quickly, and I should always be alert. If I’m not alert, a disaster may occur. It’s somehow caused me to always be anxious that something may happen” (P # 3).*

Workplace violence against nurses is another source of stress that nurses confront. In this regard, the experience of one of the participants was as follows:

*“It happened to me in the psychiatric ward; one of the patients attacked me, and because he was much bigger than me, he beat me so badly that I was injured, and despite wounded hands and feeling awful, I had to end that shift” (P # 8).*

Another condition that makes the nursing job tense is the fear of transmission of the disease and the risk of getting a needlestick:

*“It was around this time last year that I got a needlestick from a patient who was addicted, with her full body tattooed. When that happened, I was shocked, and I was completely shattered in that shift” (P # 5).*

### Occupational burnout

The nurse’s dislike of and disinterest in their profession and field affect the balance of their presence. During their presence, nurses constantly tend to reduce the time of their presence; their commitment to the field decreases; they are annoyed and do not pay attention to work:

*“When you’re not interested in your field of study, your attachment to your work decreases. You just like to do something else in the middle of work, for example, instead of taking care of the patient, you like to talk to your colleague about the house price, or check your phone ...” (P # 17).*

Due to their occupational burnout, some participants described their presence as being in a place they did not belong:

*“I didn’t have the spirit to work in the burn ward, but I had to because I wasn’t favored. I wasn’t cut out for that ward, but I was there” (P # 9).*

### Multitasking

Performing multiple tasks in the nursing profession and other activities that are not in the framework of patient care may provide grounds for energy deviation in the nurse:

*“To be honest, checking the phone, surfing the web, using telegram and WhatsApp, all distract you at work. In the middle of work, you have a feeling that you like to check your phone. Or, for example, if my phone is in my closet, I’d like to go as soon as possible to see if there’s a message for me or not” (P # 3).*

Another participant talked about studying while working:

*“When I was studying my MS, I was working at the same time. The worst memories of my shift were when I was on a shift, and I had an exam the next day. I couldn’t study at all in the ward; on the other hand, I was completely stressed for the exam” (P # 3).*

### Impaired communication

When communication is impaired, signs of contradiction, conflict, violence, mistrust, disrespect, and disagreement develop:

*“I was in a ward where my co-workers didn’t value me and had an argument with me. In this environment, it’s really hard to work. Your mind is always occupied. Instead of focusing on your job, you should spend your energy on resolving disputes” (P # 2).*

Sometimes, patient violence influences the effective presence of the nurse. In this regard, the experience of one of the participants was as follows:

*“I was a triage nurse. I told one of the patients that her condition wasn’t very emergency. As soon as I said that, he punched and kicked me in the face, actually in front of my colleagues! I felt very bad and ashamed at work for almost a month.” (P # 12).*

On the other hand, the relationship between the doctor and the nurse is one of the factors that affect nurses’ presence. In case this relationship is impaired, a sense of negative emotions dominates the ward:

*“One of the doctors is really bad-tempered. Any time he comes to visit the ward, my heart rate increases; I get clumsy, and until he gets out of the ward, I’m so anxious that I don’t know what is going on!” (P # 9).*

## Discussion

According to the findings of the present study, the main antecedent of presenteeism in nurses is *the nurse without a nurse*. In other words, the nurse who has been responsible for caring for and supporting patients has now been left without a nurse. As though there is no one to nurse a nurse. The nurse needs an advocate to notify his/her rights. Their voice is nipped in the bud and is not heard by anyone. The nurse is not seen; however, provides care for the patient with limited power in an atmosphere where injustice prevails. In this regard, the study by Nouri et al. (2019) showed that nurses suffer from a sense of being overlooked. They have stated that they are not involved in decision making and asked for feedback about their problems and needs [29]. On the other hand, the professional identity of the nurse is so damaged that their devotion seems valueless for others. They are considered as attendants who merely obey the doctor’s instructions. Nurses, despite being superior in numbers, face challenges in power, justice, facilities, identity, respect, health, and comfort. Robinson (1992) introduced a theory in nursing called the “*black hole of the theory of nursing*”, which states despite the fact that nurses present astounding advances and innovations in practice, research, education, and leadership, they still have a low public profile. It seems that all nurses’ progress is drawn to a black hole and disappears. This causes the main nature of the nursing profession to be underestimated and nurses to have the lowest voice in policymaking [30].

According to the present study, the nurse works in an environment where powerlessness, injustice, inadequate awareness, threatened health, poor structural facilities, compulsory presence, impaired communication, job stress, and job frustration prevail. This conveys that as though the nurse had been forgotten; no supporting umbrella covers him/her; there needs to be a nurse to care for him/her, to educate them to increase awareness, to take care of their body and soul, to relieve physical and mental complication, to act as an advocate for them to remove powerlessness, injustice, limited structural facilities, and compulsory presence, and to act as a counselor to reduce job burnout and stress. In other words, when we used the term “the nurse without a nurse”, our emphasis was on the fact that the nurse, who had supported the patients in every way, was not supported by the leaders and other members of the health care team, (including top- and middle-level managers, doctors, etc.). Therefore, all members and leaders of the health system (not just nurses) should provide comprehensive support to nurses as a compassionate nurse.

All these factors hinder a nurse from having an efficient presence at work. Nurses’ mind and body are involved in problems that plague them constantly in the workplace, and much of their energy is consumed on resolving problems rather than on caring for the patient.

Therefore, the nurse, being left without a nurse and lacking comprehensive support, experiences presenteeism in the work environment. In this regard, Mohammadi et al. (2020), in their study, showed that the presence-oriented nurse faces an imbalance in presence. That is to say, the nurse’s cognitive, emotional, and motor capacities do not alter from potential to actual. The nurse will not have the mental processing power of the past, will not provide effective emotional care, and will not be physically able to provide service for long hours at the patient’s bedside [13]. The central reason for the lack of actualization of these capacities was revealed in the present study. The nurse, as a person who provides care for the patient’s body and soul, is not physically and mentally cared for.

The limitation of the present study was collecting the data only from two hospitals, which could create a bias associated with the policies and practices specific to those two hospitals. Therefore, it is suggested that more diverse hospitals be considered in future studies.

## Conclusion

In general, the results of the present study showed that the main antecedent of presenteeism in nursing is *the nurse without a nurse*. It means that the nurse, who has been responsible for caring, supporting, advising, advocating, and educating the patient, has now been left without anyone performing these roles for her/him. It is recommended that the results of this study be used in health policy-makings. This study helps nursing leaders and managers to better understand nursing staff in their management style and prevent the emergence of presenteeism in nurses. The results of this study can make nurses’ voices heard by health leaders and managers. A voice that has never been heard as it deserves. This study enlightens the pains of nurses and their unheard words. Besides, the results of this study can be taught to students as educational content in educational programs of nursing schools to help students gain insights from the very beginning into the conditions leading to presenteeism.

## Supplementary Information

**Additional file 1.** Demographic characteristics of participants in the qualitative study.

## Data Availability

The data analyzed and materials used in this study are available from the corresponding author on reasonable request.
