# Supplementary material for: The nurse without a nurse: the antecedents of presenteeism in nursing
Source: BMC Nurs. 2021 Aug 13;20:143. doi: 10.1186/s12912-021-00669-1 (PMC8361635; doi:10.1186/s12912-021-00669-1)
Supplement: Supplementary file 1 — Additional file 1. Demographic characteristics of participants in the qualitative study. [file 12912_2021_669_MOESM1_ESM.doc]

Additional file 1. Demographic characteristics of participants in the qualitative study

| Number | Gender | Age | Marital Status | Level of Education | work experience  (Year) | Place of interview | Interview duration |
| --- | --- | --- | --- | --- | --- | --- | --- |
| 1 | Female | 31 | Married | Bachelor | 6 | Hospital | 90 |
| 2 | Male | 33 | Single | Bachelor | 7 | Hospital | 90 |
| 3 | Male | 36 | Married | Master of Science | 12 | Hospital | 70 |
| 4 | Female | 30 | Single | Master of Science | 7 | Hospital | 60 |
| 5 | Female | 32 | Married | Bachelor | 9 | Hospital | 60 |
| 6 | Male | 34 | Married | Bachelor | 9 | Hospital | 60 |
| 7 | Female | 35 | Married | Bachelor | 10 | Hospital | 90 |
| 8 | Female | 39 | Married | Bachelor | 13 | Hospital | 60 |
| 9 | Female | 45 | Married | Bachelor | 21 | Hospital | 75 |
| 10 | Male | 48 | Married | Bachelor | 23 | Hospital | 60 |
| 11 | Female | 37 | Single | Bachelor | 14 | Hospital | 90 |
| 12 | Female | 40 | Married | Bachelor | 17 | Hospital | 90 |
| 13 | Male | 52 | Married | Bachelor | 28 | Hospital | 45 |
| 14 | Female | 30 | Single | Bachelor | 6 | Hospital | 60 |
| 15 | Female | 32 | Married | Bachelor | 8 | Hospital | 60 |
| 16 | Male | 33 | Single | Bachelor | 9 | Hospital | 70 |
| 17 | Male | 47 | Married | Master of Science | 22 | Hospital | 60 |
